# Lessons from History for Designing and Validating Epidemiological Surveillance in Uncounted Populations

**DOI:** 10.1371/journal.pone.0022897

**Published:** 2011-08-03

**Authors:** Peter Byass, Osman Sankoh, Stephen M. Tollman, Ulf Högberg, Stig Wall

**Affiliations:** 1 Department of Public Health and Clinical Medicine, Umeå Centre for Global Health Research, Umeå University, Umeå, Sweden; 2 MRC/Wits Rural Public Health and Health Transitions Research Unit (Agincourt), School of Public Health, Faculty of Health Sciences, University of the Witwatersrand, Johannesburg, South Africa; 3 INDEPTH Network, Accra, Ghana; 4 Department of Women's and Children's Health, Uppsala University, Uppsala, Sweden; Institute for Clinical Effectiveness and Health Policy (IECS), Argentina

## Abstract

**Background:**

Due to scanty individual health data in low- and middle-income countries (LMICs), health planners often use imperfect data sources. Frequent national-level data are considered essential, even if their depth and quality are questionable. However, quality in-depth data from local sentinel populations may be better than scanty national data, if such local data can be considered as nationally representative. The difficulty is the lack of any theoretical or empirical basis for demonstrating that local data are representative where data on the wider population are unavailable. Thus these issues can only be explored empirically in a complete individual dataset at national and local levels, relating to a LMIC population profile.

**Methods and Findings:**

Swedish national data for 1925 were used, characterised by relatively high mortality, a low proportion of older people and substantial mortality due to infectious causes. Demographic and socioeconomic characteristics of Sweden then and LMICs now are very similar. Rates of livebirths, stillbirths, infant and cause-specific mortality were calculated at national and county levels. Results for six million people in 24 counties showed that most counties had overall mortality rates within 10% of the national level. Other rates by county were mostly within 20% of national levels. Maternal mortality represented too rare an event to give stable results at the county level.

**Conclusions:**

After excluding obviously outlying counties (capital city, island, remote areas), any one of the remaining 80% closely reflected the national situation in terms of key demographic and mortality parameters, each county representing approximately 5% of the national population. We conclude that this scenario would probably translate directly to about 40 LMICs with populations under 10 million, and to individual states or provinces within about 40 larger LMICs. Unsubstantiated claims that local sub-national population data are “unrepresentative” or “only local” should not therefore predominate over likely representativity.

## Introduction

Because of the relative scarcity of reliable individual data at the national population level, particularly in Africa and Asia, many national and global estimates of population health have to rely on extrapolation and modelling approaches using the few available data [1], even though such estimates have been described as “a necessary evil” [2]. National data in low and middle income countries (LMICs) are often of dubious quality and validity, while quality data from discrete local areas may be considered to lack representativity. While the long term aim must be to work towards better quality national data [3], it is important to consider interim strategies, such as making the best use of more local data [4]. One source of population health data is provided by localised health and demographic surveillance systems (HDSSs) in Africa and Asia, such as those affiliated to the INDEPTH Network [5], in which geographically defined populations in particular localities are followed in detail on a longitudinal basis. This approach can yield rich and detailed data, which are otherwise completely unavailable in many countries on a national scale. These data, at the individual level, are comparable in quality to corresponding national data in well-established settings such as Scandinavia, but only relate to discrete populations in relatively small areas. Consequently, the criticism is repeatedly raised that such data are “only local” or “unrepresentative”, a viewpoint that is hard to refute in absolute terms in LMICs because of the *a priori* lack of reliable data on a wider scale for comparison.

Conventional statistical theory is not particularly useful for addressing this question, in the absence of reliable data from populations outside surveyed areas. To some extent it is possible to compare HDSS data with such other data as are available, for example from cross-sectional Demographic Household Survey (DHS) data or other censuses and surveys. This has been done in a small number of previous studies. A comparison of HDSS data on childhood mortality from Butajira in Ethiopia with two rounds of DHS birth history data showed similar mortality rates over time, despite the inherent recall problems of the DHS approach [6]. A further study from Butajira assessed possible differences in mortality risk factors between HDSS and DHS sources [7]. At Matlab in Bangladesh a comparison was made between DHSS and DHS approaches to reproductive health data [8]. The Nouna HDSS in Burkina Faso has also been compared with DHS data [9], and in Mozambique a three-way comparison between HDSS, DHS and national census data on mortality has been made [10]. However, all of these comparisons are less than definitive, because at best they make comparisons with sampled national data that are not demonstrably more reliable than the HDSS data themselves, and usually less detailed.

In this paper we have taken a novel and alternative approach to empirically demonstrating the representative potential of epidemiological data from one locality within a country. To start with, we looked for possible sources of detailed national data, with complete individual registration including age-, sex- and cause-specific mortality, but with patterns of demographic and epidemiological characteristics reasonably similar to those in many currently uncounted populations. No LMICs currently have such data with complete population coverage. Contemporary data from industrialised countries were excluded on the grounds of demographic and socioeconomic incomparability, leaving the possibility of using historic data from an enumerated population that could be more comparable with contemporary situations in Africa and Asia. Scandinavian countries have long traditions of detailed individual registration, and we explored using historic Swedish national statistics as the basis for these empirical investigations. Our starting point was to address the question “when did Sweden have a demographic and socioeconomic profile that could be described as an LMIC in today's terminology?”.

Our aim, after demonstrating reasonable demographic and socioeconomic comparability between the historic data and contemporary population of the developing world, was to take national level figures from the chosen population as the “gold standard” for comparison with local area figures from the same population, in order to see what proportion of individual local areas might have served as reliable proxies for the national results, had their local populations been chosen to be followed in an “HDSS” mode of operation. We then interpreted these findings in relation to contemporary LMIC populations.

## Methods

**Figure 1:**
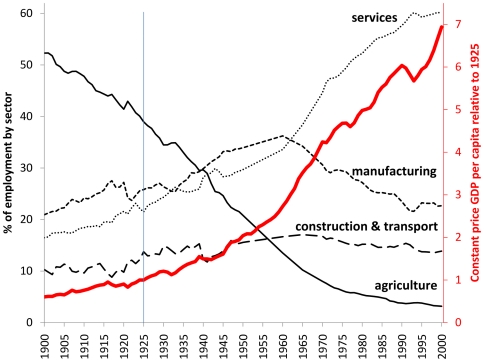
Key aspects of Sweden's developmental trajectory during the 20^th^ century.

**Figure 2:**
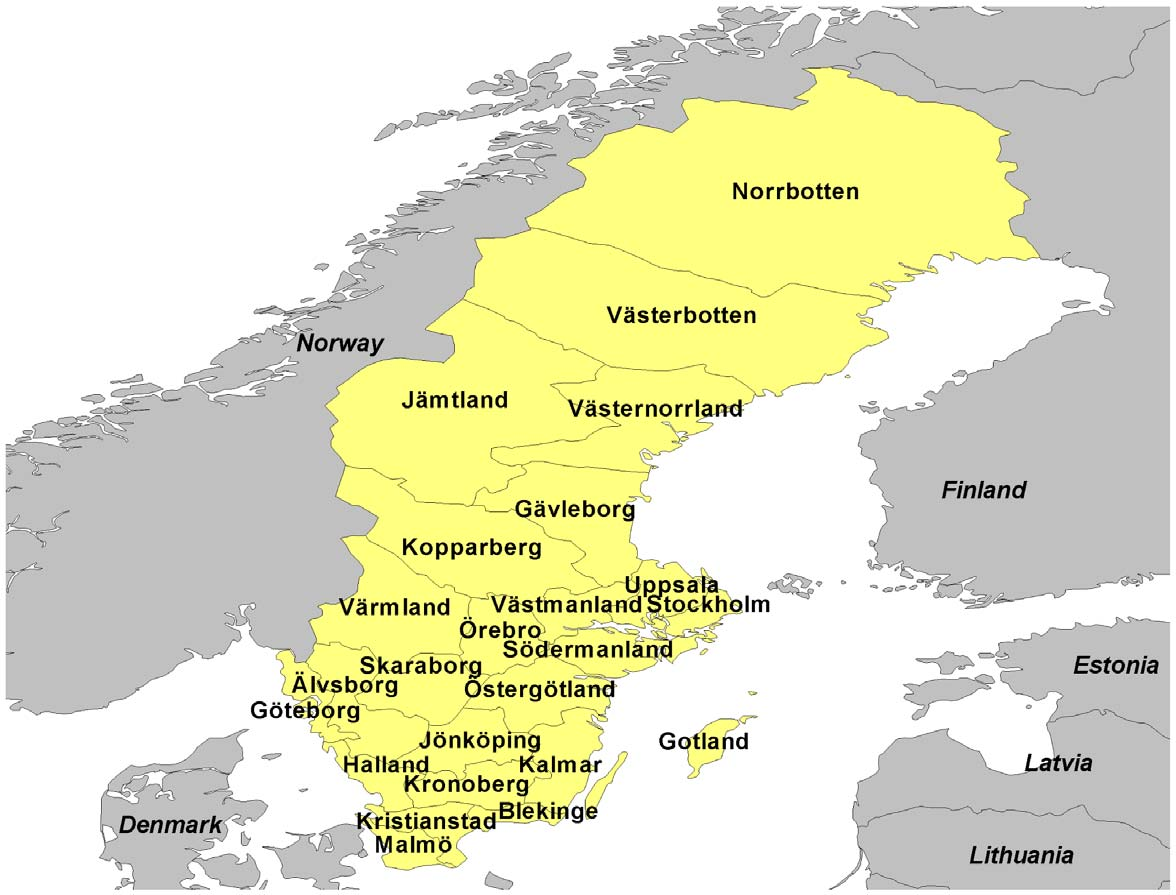
Map of Sweden, showing counties.

We carried out an initial assessment of historic Swedish population data to find a point in Sweden's epidemiological transition where basic population and socioeconomic characteristics were similar to today's LMIC population. Figure 1 illustrates key aspects of Sweden's developmental trajectory during the 20^th^ century, moving rapidly away from a traditional agrarian society into a mid-period of manufacturing dominance and later emphasis on the service sector [11], [12]. The century saw a more than tenfold increase in per capita GDP when calculated on a constant price basis, meaning that per capita income for Swedes in 1925 was equivalent to approximately US $3,900 in modern terms [13]. We selected 1925 as a comparable point in relation to contemporary LMICs, being in the inter-war period (even though Sweden maintained neutrality in both World Wars and was thus less affected than other nations) and at a point of rapid developmental transition. Swedish official statistics for 1925 [14], [15] then provided cause of death data and other demographic details nationally and by county (the highest level of sub-national administration in Sweden), which form the primary source for these analyses. Sweden's population in 1925 was 6,053,562, organised in 24 counties as shown in Figure 2. Urban Stockholm was administered separately from surrounding Stockholm county at that time, but the combined figures for the overall county have been used here. The average population per county was thus 252,232, around the same order of magnitude as many contemporary HDSS populations. County populations ranged from 704,531 for Stockholm to 56,981 in the island county of Gotland.

Causes of death from the Swedish 1925 data were consolidated slightly from the categories in which they were recorded, so that “old age” and “undetermined” were combined into a single “undetermined” category, and several smaller specific categories (psychiatric, musculo-skeletal, blood, endocrine, and skin disorders) combined into an “other” category, resulting in 12 overall cause of death categories for further analysis. It should be noted that the category “nervous system” included cerebrovascular events, as distinct from “circulatory” causes. Population-based rates were calculated for each cause group, at national and county level.

Other population rates for Sweden in 1925 (births, stillbirths, infant and maternal deaths) were calculated at national and county levels.

No ethical approval was required for this study which was solely based on published historical data.

## Results

**Figure 3:**
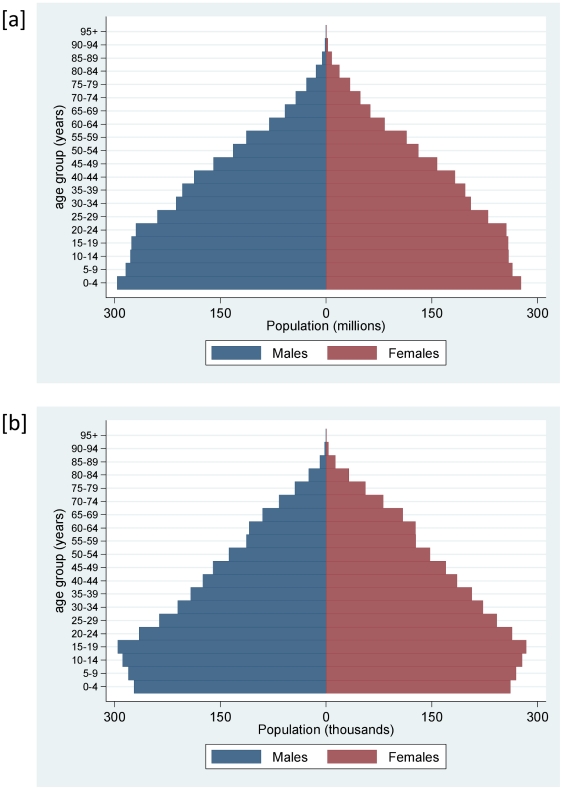
Population structures for [a] population of less developed countries in 2010 and [b] Sweden in 1925.

The age-sex breakdown of the Swedish national population for 1925 is shown in Figure 3, in comparison with the current-day UN population estimates for less-developed countries [16]. The similar shapes of these pyramids indicate populations at a similar stage of epidemiological transition, the main difference being declining fertility in Sweden, indicated by the slightly lower proportions of population in the lower age groups. Using a variety of sources [14]–[19], Table 1 shows comparisons of basic demographic and epidemiological parameters on a similar basis.

**Table 1:** Comparison of key parameters between the Swedish population of 1925 and the current population of low- and middle-income countries.

parameter		current LMICs	Sweden in 1925
crude birth rate	per 1,000	21.2	17.7
total fertility rate	per lifetime	2.62	2.34
life expectancy at birth	years	67.0	62.6
maternal mortality ratio	per 100,000 live births	290	261
infant mortality rate	per 1,000 live births	49	55
under-5 mortality rate	per 1,000	72	73
dependency ratio	(under 15 y + over 65 y)/15–65 y	0.54	0.56
overall crude mortality rate	per 1,000	9.3	11.7
cause-specific mortality fractions	infection	16.8%	16.3%
	circulatory	18.1%	16.9%
	respiratory	13.7%	9.2%
	cancer	10.2%	11.1%
	nervous system	9.3%	11.7%

**Figure 4:**
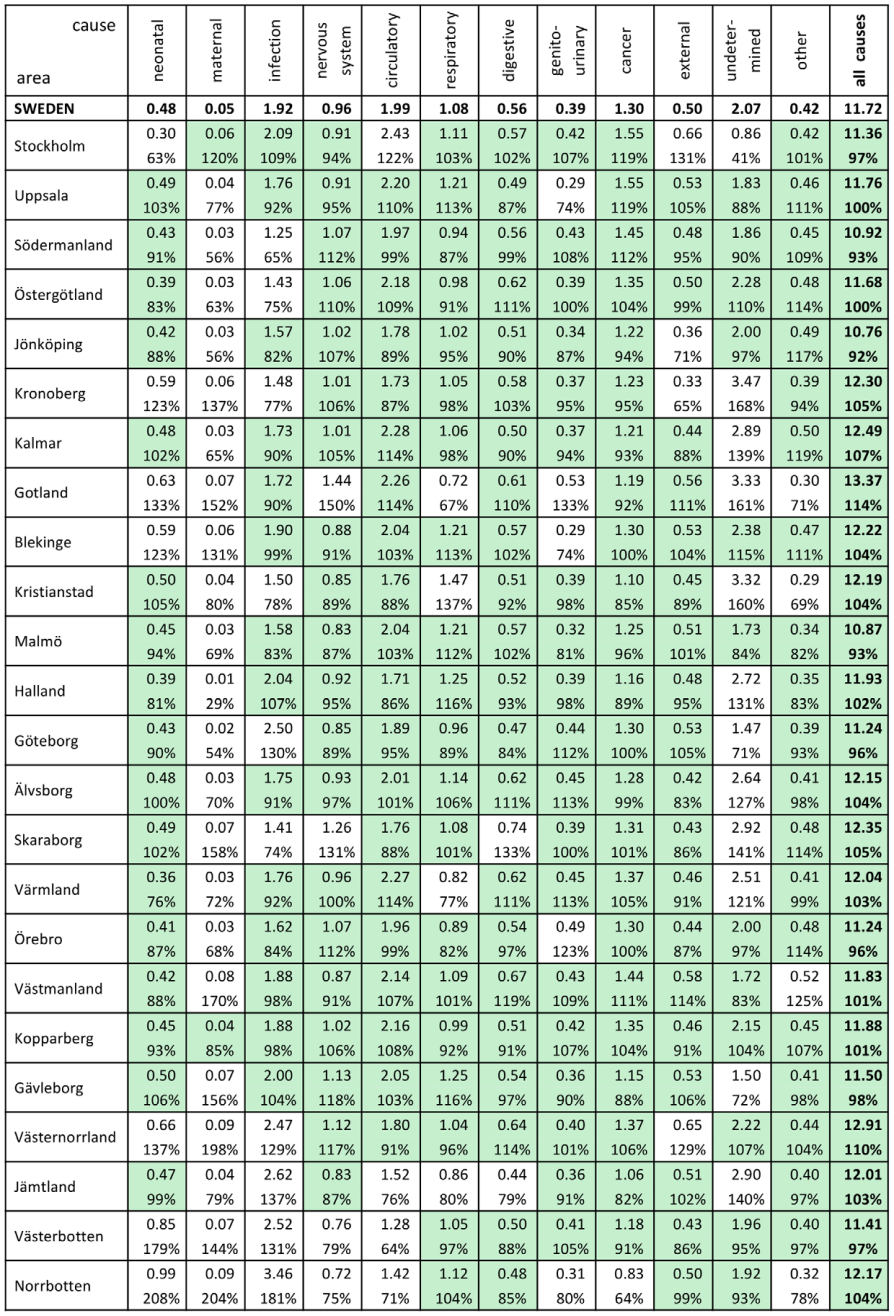
Cause-specific mortality rates for Sweden in 1925, nationally and by county. Percentages represent proportions of national rates, for 12 mutually exclusive cause of death categories and for overall mortality. Shaded cells indicate county results within ±20% of national values.

**Figure 5:**
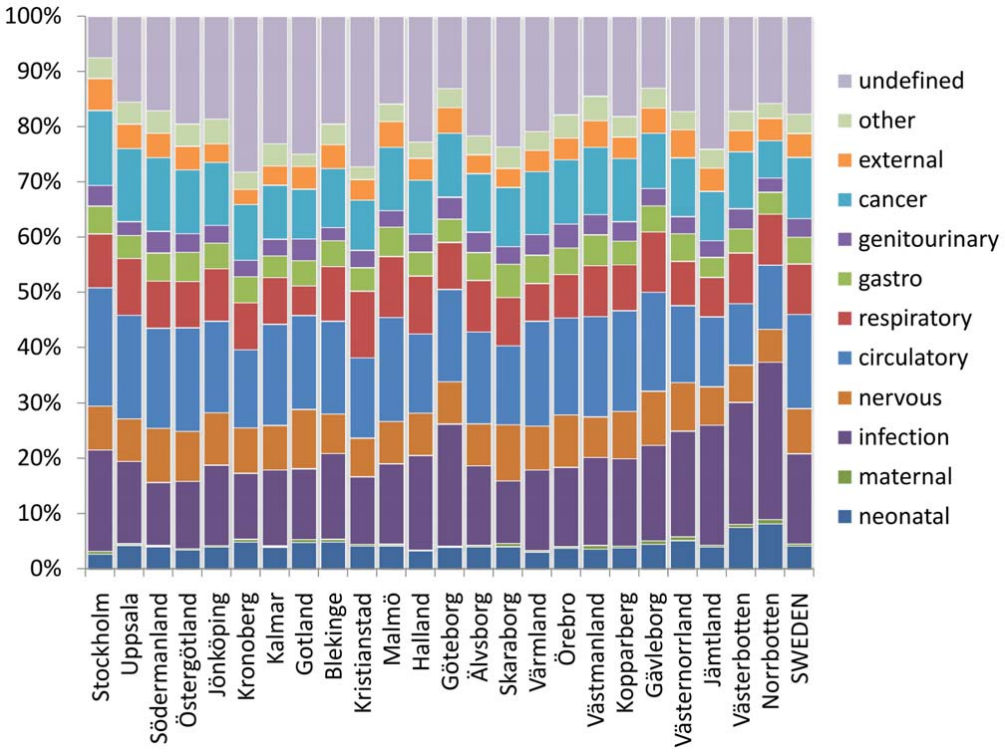
Cause-specific mortality fractions for Sweden, 1925, by county and at national level.

Overall mortality rates by county were within ±10% of the national rate, 11.72 per 1,000, except for the county of Gotland (114% of the national rate), and Figure 4 shows cause-specific mortality rates (per 1,000) by county and nationally, also showing county rates as percentages of the corresponding national rates and indicating which county rates (shaded cells) lay within ±20% of the national equivalent. The counties of Stockholm, Gotland, Jämtland, Västerbotten and Norrbotten showed the greatest deviations from national rates. The only specific cause for which the majority of county values varied by more than 20% was maternal mortality, because of the relatively small numbers of such deaths per county. Figure 5 shows cause-specific mortality fractions by county and for Sweden as a whole. Stockholm showed relatively higher rates for cardiovascular, cancer and external-cause mortality, coupled with a lower rate of undetermined causes. Northernmost counties showed relatively higher rates of infection, with lower rates of cardiovascular and cancer mortality. Gotland, despite its higher absolute mortality rate, did not show a markedly different pattern by cause.

Table 2 shows a more public-health oriented approach to the same data, with the top five causes of mortality being ranked for each county and at national level. Nationally the top five ranked causes were undetermined, circulatory, infection, cancer and respiratory, together accounting for 71% of total mortality. Only six particular causes featured among the top five causes across all counties and nationally (undetermined, circulatory, infection, cancer, respiratory and nervous system).

**Table 2:** Top five ranked causes of mortality for Sweden nationally in 1925 and by county.

	ranked cause				
area	first	second	third	fourth	fifth
**SWEDEN**	**undetermined**	**circulatory**	**infection**	**cancer**	**respiratory**
Stockholm	circulatory	infection	cancer	respiratory	nervous system
Uppsala	circulatory	undetermined	infection	cancer	respiratory
Södermanland	circulatory	undetermined	cancer	infection	nervous system
Östergötland	undetermined	circulatory	infection	cancer	nervous system
Jönköping	undetermined	circulatory	infection	cancer	nervous system
Kronoberg	undetermined	circulatory	infection	cancer	respiratory
Kalmar	undetermined	circulatory	infection	cancer	respiratory
Gotland	undetermined	circulatory	infection	nervous system	cancer
Blekinge	undetermined	circulatory	infection	cancer	respiratory
Kristianstad	undetermined	circulatory	infection	respiratory	cancer
Malmö	circulatory	undetermined	infection	cancer	respiratory
Halland	undetermined	infection	circulatory	respiratory	cancer
Göteborg	infection	circulatory	undetermined	cancer	respiratory
Älvsborg	undetermined	circulatory	infection	cancer	respiratory
Skaraborg	undetermined	circulatory	infection	cancer	nervous system
Värmland	undetermined	circulatory	infection	cancer	nervous system
Örebro	undetermined	circulatory	infection	cancer	nervous system
Västmanland	circulatory	infection	undetermined	cancer	respiratory
Kopparberg	circulatory	undetermined	infection	cancer	nervous system
Gävleborg	circulatory	infection	undetermined	respiratory	cancer
Västernorrland	infection	undetermined	circulatory	cancer	nervous system
Jämtland	undetermined	infection	circulatory	cancer	respiratory
Västerbotten	infection	undetermined	circulatory	cancer	respiratory
Norrbotten	infection	undetermined	circulatory	respiratory	cancer

**Figure 6:**
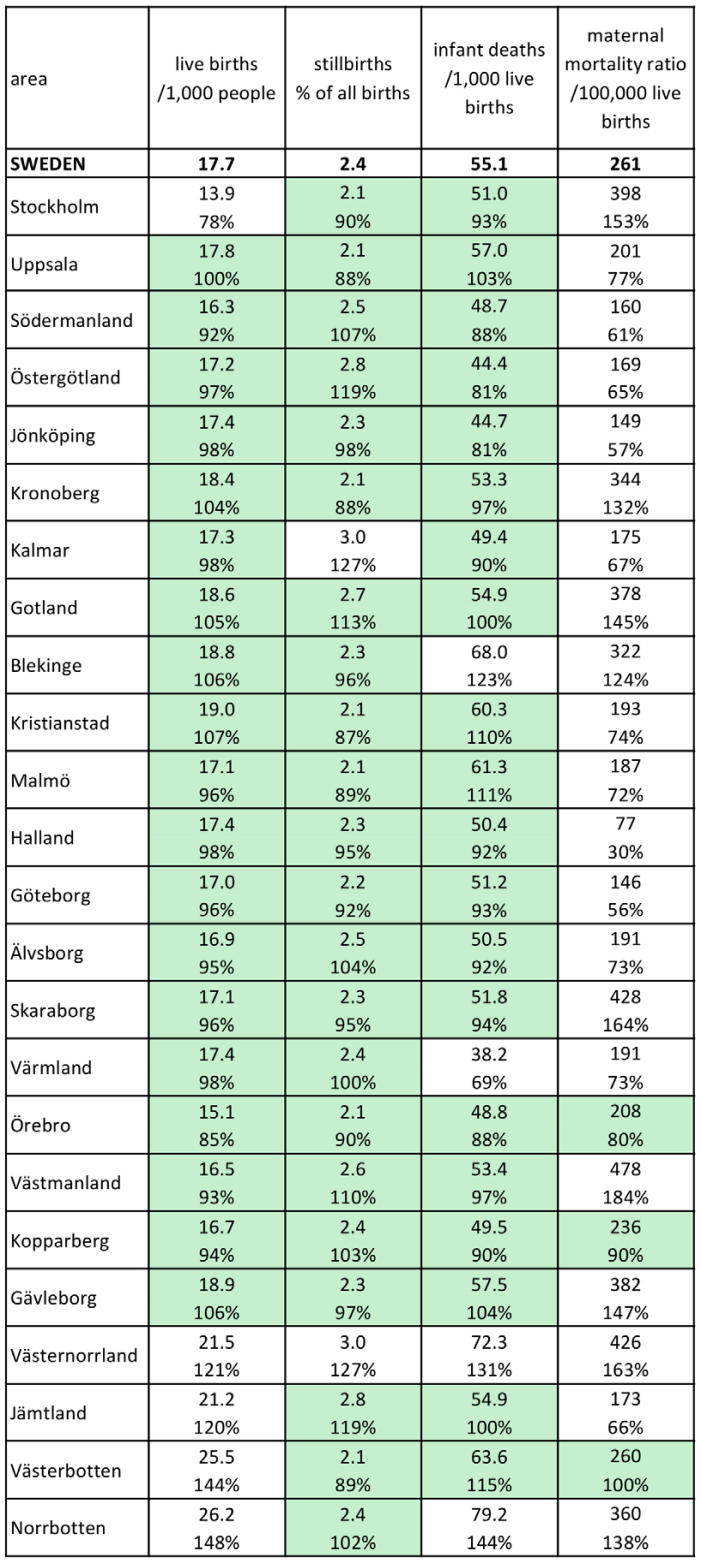
Demographic parameters for Sweden in 1925, nationally and by county. Percentages represent proportions of national rates, for live births, stillbirths, infant deaths and maternal mortality. Shaded cells indicate county results within ±20% of national values.

Figure 6 shows key demographic parameters (live birth rate, stillbirth proportion, infant death rate and maternal mortality ratio (MMR)) for Sweden in 1925, nationally and at county level. With few exceptions apart from the remote northern counties, live birth, stillbirth and infant death rates at county level were closely similar to national values, almost all within ±20% (shaded cells) and mostly within ±10%. As was the case with maternal mortality rates by county, MMR by county generally varied by more than ±20%.

## Discussion

These analyses clearly demonstrate that any one of the majority of Swedish counties in 1925 could have acted as reliable proxies for national mortality patterns, had they been used as local surveillance sites in the absence of national registration. What are the practical consequences of this finding? The consequences depend on firstly establishing the relative comparability of the Swedish population in 1925 with the current situation in the developing world, as an important link in being able to translate the historic Swedish situation into present day populations. Given that comparability, how can the 1925 Swedish pattern of sub-national differences be interpreted in terms of present-day countries or regions with local population datasets but without complete national registration processes?

### Relationships between mortality patterns at national and county level in Sweden during 1925

Apart from the five counties previously mentioned as deviating from the national figures (Stockholm, Gotland, Jämtland, Västerbotten and Norrbotten), the remaining 19 (79%) showed patterns strongly similar to the Swedish nation as a whole, in terms of cause-specific mortality rates, cause-specific mortality fractions and leading causes of death. Possible reasons as to why the five less representative counties may have differed from national levels are not hard to find, and they seem to constitute a non-random group. Stockholm included the capital city, and the observed differences included higher rates of deaths from circulatory and external causes. The appreciably lower level of undetermined causes in Stockholm may reflect different patterns of death certification and health service utilisation. Gotland is a unique county in that it is not part of mainland Sweden, but an island in the Baltic Sea. Although its cause specific mortality fractions were similar to the national picture, it had the highest overall mortality rate. The other three discrepant counties comprised a contiguous block of very sparsely populated territory in the northernmost part of the country, including a considerable area lying to the north of the Arctic Circle. These three counties had a population density of 2.4 km^−2^ compared with the national figure of 13.6 km^−2^. The higher rates of deaths from infectious and neonatal causes in these counties are thus not surprising, consistent also with the higher birth and infant death rates observed. Maternal mortality was deliberately left as a single cause in our analyses, because of its public health significance. Even though the overall maternal mortality ratio was 261 per 100,000, this only amounted to around 10 maternal deaths per county during the year, a level which resulted in relatively large random variation and hence a lack of concordance (both for maternal mortality as a single cause and MMR) with the national figure, illustrating the inherent difficulty of measuring relatively rare events on a population basis.

### Sweden in 1925 as a model for current LMIC populations

Clearly there are many similarities between the historic Swedish population and current LMIC populations, but also important differences. In terms of its overall demographic and socioeconomic profile, Sweden in 1925 would in contemporary parlance be designated as an LMIC. However, in 1925 the absence of HIV/AIDS and the non-availability of vaccines and antibiotics could represent important differences. On a wider scale, factors relating to geography, climate, governance, politics and economics could be considered as major disparities, but equally contemporary LMICs are not all equivalent in these respects. The rapid developmental transition away from agricultural dominance seen in Sweden in 1925 was similar in many respects to current developments in various LMICs. Similarly, the equivalences in population structure and, according to GBD estimates, mortality patterns, as shown in Table 1 and Figure 3, indicate that these are populations at closely comparable stages of epidemiological transition. Demographic and epidemiological changes in populations are inherently gradual processes that proceed over decades, and are therefore not strongly influenced by shorter-term circumstances. Both in present-day LMICs and in Sweden in 1925, populations were characterised by relatively high mortality, arising from a mixture of infectious and chronic causes, and a relatively small proportion of older people. Thus, at least in terms of demographic and epidemiological factors, there are strong grounds for claiming substantial comparability between these populations. In addition, the quality of data in the 1925 Swedish national data, including some characteristics such as a relatively high proportion of deaths from undefined causes, appears comparable to the quality achieved within contemporary local HDSS populations.

Although in principle one might wish to undertake similar analyses in present-day LMICs, even countries which now considerably out-perform Sweden in 1925 in economic and demographic terms generally lack the completeness and quality of vital registration data needed for this approach. To take the example of Thailand, now classified by the World Bank as an upper middle-income country, considerable shortcomings in the quality of national data were identified in a careful evaluation [20], [21].

### Translating the Swedish findings to other populations

Having established comparability at the population level, the more important issue to consider is how the Swedish local area data (at county level) in relation to its national “gold standard” can be interpreted in terms of present-day LMIC local area data (which usually have no reliable national standard for comparison or validation). Given that 19/24 of the Swedish counties yielded results which were closely representative of the national picture, one might argue that one area selected at random would stand an 80% chance of being representative. More constructively, given that the less representative counties had self-evident characteristics (capital city, not part of the mainland, remote and sparsely populated) likely to contribute to the observed differences, it is more helpful to argue that any single county could be representative, provided obvious outliers were excluded.

However, the concepts of country and county need to be explored in order to translate the historic Swedish findings more widely. Countries of the world vary hugely in both physical and demographic size, with different arrangements for administrative sub-areas, and clearly do not constitute an ideally uniform unit of epidemiological observation [22]. The Swedish findings, from a time when Sweden was effectively an LMIC, related to a population of around 6 million comprised of local areas amounting to approximately 5% of the total, which could be translated directly to around 40 of the world's developing countries with populations of the order of 1 to 10 million. For the 40 or so larger developing countries, it would probably be more reasonable to argue for translating the Swedish findings at sub-national level, so that one population sub-unit within a particular state or province (for example in India or China) might be considered as representative of that state or province. These conclusions have important implications for analyses that might use data from one or several HDSS populations [23].

### Conclusions

Among populations undergoing epidemiological transition, with relatively high mortality and a fairly low proportion of older people, we conclude from the equivalent historic Swedish data that most local areas (comprising around 5% of the total population) can yield population data which are representative of a total population of up to 10 million. Nevertheless it is important to exclude obvious outliers in choosing a representative area: for example, major cities, or remote and isolated regions. In view of these findings, unsubstantiated claims that local area population data are “only local” or “unrepresentative” in relation to surrounding areas should not therefore by default take precedence over likely representativity. Our findings provide an indirect validation to the principle that most detailed local area data, such as those produced by HDSS centres, are likely to be adequately representative of national data and hence suitable for generalising into policy. This conclusion is of major importance for national settings where a scarcity of data inhibits evidence-based policy development, and where all too often good analyses of local data are ignored on the grounds of being non-national.
